# Dietary behavior and urinary gallic acid concentration differences among underserved elder racial and ethnic minorities in New York City

**DOI:** 10.1007/s10552-022-01581-y

**Published:** 2022-04-19

**Authors:** Cristina N. Zambrano, Wenyue Lu, Cicely Johnson, Maayan Beeber, April Panitz, Safa Ibrahim, Marilyn Fraser, Grace X. Ma, Khursheed Navder, Ming-Chin Yeh, Olorunseun O. Ogunwobi

**Affiliations:** 1 Department of Biological Sciences, Hunter College of the City University of New York, New York, USA; 2 Hunter College Center for Cancer Health Disparities Research (CCHDR), Hunter College of the City University of New York, 695 Park Avenue, HN310A, New York, 10065 USA; 3 Center for Asian Health, Lewis Katz School of Medicine, Temple University, Philadelphia, PA USA; 4 Sociology Department, College of Liberal Arts, Temple University, Philadelphia, PA USA; 5 Nutrition Program, School of Urban Public Health, Hunter College of the City University of New York, New York, USA; 6 Arthur Ashe Institute for Urban Health, Brooklyn, New York, USA; 7 Department of Clinical Sciences, Lewis Katz School of Medicine, Temple University, Philadelphia, PA USA

**Keywords:** Cancer prevention, Minority, Nutrition, Antioxidants, Fruits and vegetables

## Abstract

**Purpose:**

Diet and nutrition are important for cancer prevention. To investigate associations between dietary behavior, demographics, and risk of cancer, we assessed dietary behavior and urinary concentration of gallic acid, a polyphenol with anticancer properties found in various fruits and vegetables, in racial and ethnic minorities.

**Methods:**

Ninety-one (91) participants were recruited from senior centers in East Harlem, New York City, a racially diverse and underserved community. A National Institute of Health (NIH)—validated dietary survey questionnaire—was used to collect dietary fruits and vegetables consumption data. Demographic and cancer information were also collected. All 91 participants completed the survey and forty-five (45) participants provided urine samples for gallic acid analysis.

**Results:**

Gender differences were significantly associated with dietary behavior and urinary gallic acid concentration (UGAC). Female participants had a higher total daily intake of fruits and a significantly higher UGAC compared to male participants (*p* < 0.05). Age was negatively associated with the serving quantity of French fries/fried potatoes and white potatoes (*p* < 0.05), while positively associated with the daily intake frequency and daily intake of fruits (*p* < 0.05). Furthermore, Asian race was associated with higher daily intake frequencies of fruits and vegetable soup (*p* < 0.05), compared to other races. In a multivariate analysis, a significant association was observed between the serving quantities of fruits and other vegetables and UGAC (*p* < 0.05) after controlling for demographic characteristics.

**Conclusion:**

The observed differences in dietary behavior and UGAC in this study provide limited information on the association between demographic differences and cancer prevalence in elder racial and ethnic minorities. Future research should investigate this association further for potential implications in cancer prevention.

**Supplementary Information:**

The online version contains supplementary material available at 10.1007/s10552-022-01581-y.

## Introduction

Cancer is the second leading cause of death in the United States [1]. While cancer affects all groups in the United States, cancer disparities disproportionately affect some groups compared to others [2]. Black/African-Americans have a higher incidence and mortality rate of all cancers compared to other racial/ethnic groups in the United States [3]. Hispanics/Latinos have a higher incidence of late-stage colorectal cancer at an earlier age and poorer age adjusted survival compared to non-Hispanic Whites [4]. Furthermore, men have higher incidence and mortality rates compared to women [5]. In addition, income can also affect cancer rates. People living in low-income and middle-income counties have higher chances of dying from cancer than those living in high-income counties [6].

Additionally, some cancers have higher incidence in relation to specific demographic factors. For example, colorectal, stomach, liver, and pancreatic cancers are more common among men than women [7–10]. Genetic mutations and biological differences have been shown to contribute to higher incidence of certain cancers in men than women [11, 12], and among Black/African-Americans and Hispanics compared to Non-Hispanic Whites [13, 14]. However, there are also external factors that can contribute to cancer disparities. Diet plays a significant etiological role in cancer [15]. Certain cancers such as colorectal, stomach, and liver are associated with diet [16–18]. A high-fat diet has been linked to increased risk of cancer [19], while a diet rich in fruits, vegetables and fiber can potentially reduce the cancer risk [20].

In this study, we assessed the dietary behavior and urinary gallic acid concentration (UGAC) of residents in East Harlem, an underserved neighborhood in New York City. We investigated the association between dietary behavior and UGAC, in relation to cancer risk. We used a combination of Food Frequency Questionnaires (FFQ), which are widely used dietary self-assessments [21], in conjunction with the urinary biomarker gallic acid, which is an objective measure of fruit and vegetable intake, to obtain more accurate and comprehensive information [22]. Gallic acid (3,4,5, trihydroxybenzoic acid) is a dietary polyphenol shown to have important anticancer properties [23–25]. As a stable polyphenol, gallic acid is efficient as a unit of measurement for polyphenol quantification [26].

Previous studies have investigated dietary behaviors associated with cancer prevention in young people [27, 28]. However, there are notable gaps in knowledge and lack of nutritional research in the elderly population affected by cancer [29], as well as scarce biospecimen availability from diverse populations, limiting cancer health disparities research [30]. In this study, we aimed to explore dietary behavioral differences among diverse elder residents of East Harlem, New York City and associations to cancer risk, to understand whether these differences can provide insights into cancer prevention strategies that will have impact in addressing cancer health disparities in racial and ethnic minorities.

## Materials and methods

### Participants

Study participants were recruited by Collaborative Institutional Training Initiative (CITI) certified research assistants at senior centers, managed by Union Settlement. The study was approved by the Institutional Review Board (IRB) at The City University of New York. Nine-one (91) participants completed the demographic information and dietary survey, while forty-five (45) participants also provided urine samples. The age of all participants ranged between 54 and 87 years old, with a mean age of 72.3 years.

The study team informed and consented participants before any information was collected from them. ID codes were assigned to participants on their survey forms and urine samples. No incentive or payment was provided to the enrolled participants.

### Data collection

A seven-item questionnaire was used to measure demographics. In the questionnaire, information regarding gender, age, race/ethnicity, marital status, education, and income was collected. To assess dietary behavior, we used the FFQ from the Eating at America’s Table Study (EATS) [31], as previously described [32]. In summary, dietary information regarding various foods including fruits, vegetables, and beverages was collected. Furthermore, urine samples of the consenting participants were collected on site to determine UGAC. The samples were transported in coolers back to the laboratory for analysis. If the participants did not consent to urine storage, the sample was discarded after analysis. If consented, each sample was aliquoted and stored at -80 °C for future analysis [32].

### Analysis of urinary gallic acid concentration (UGAC)

Analysis of UGAC was performed adapting the Fast Blue 4-benzoylamino-2,5-diethoxybenzenediazonium chloride hemi salt (FBBB) method. Briefly, a gallic acid standard curve with a range of dilutions (0, 25, 50, 100, 250, and 50 µg/mL) was generated. Each urine sample was diluted to 25 µL in 975 µL of water. The standards and samples were incubated for 90 min at room temperature after the addition of 100 µL of 0.1% solution of FBBB and 400 µL of 5% sodium hydroxide. Absorbance was measured to quantify the gallic acid concentration in the urine samples at 420 nm using a Spectra Max i3 at 37 °C [32].

### Statistical analysis

To examine the associations among demographic predictors, dietary behavior, and UGAC, we conducted bivariate analysis using correlation for continuous variables, and *t* test and ANOVA for categorical variables and continuous variables, as previous stated [32]. The dietary information was analyzed as total daily intake, daily intake frequency, and serving quantity. Using the 2005 myPyramid calculation, total daily intake was obtained by multiplying the daily frequency and the serving quantity. Intake frequency was standardized as the times per day each item was consumed. Serving quantity was calculated as cup equivalents and measured serving cup size of each item, every time it was consumed. We conducted all statistical analysis with SPSS 21.0. Tests of statistical significance and 95% CIs were two-sided. A *p* value lower than 0.05 is considered statistically significant, and a *p* value lower than 0.1 is considered as marginally significant to detect potential significant trends.

## Results

### Demographics

The mean age of all participants was 72.3, with a standard deviation of 8.29 (Table 1). Most participants self-identified as female (82.2%), and reported an income lower than $50,000 (85.3%). Slightly less than half of them self-identified as Hispanic/Latino (45.1%) and had high school or lower education (48.3%). With regard to marital status, only more than one fifth of the participants were married (23.3%). Among all recruited participants, two male and one female participant reported cancer incidence (Table 1). Participants identified as Hispanic/Latino, Black/African-American and White. Two of them reported being widow and one married. Additionally, one reported to have completed high school, one some college and the other completed graduate school. All three participants with cancer prevalence reported an annual income lower than $50,000. Demographics between participants who provided urine samples were compared to demographics of participants who did not provide urine samples (Table 1). Notably, there was a significantly higher percentage (*p* < 0.05) of female participants who did not provide urine samples (91.1%) compared to the percentage of female participants who provided urine samples (73.3%). No other significant differences were identified between participants who provided urine samples and participants who did not provide urine samples.

**Table 1:** Participants’ demographics

	All cases (*N* = 91)		Cancer cases (*N* = 3)	
	Frequency/mean	Percent (%)/SD	Frequency/mean	Percent (%)/SD
Gender				
Male	16	17.8	2	66.7
Female	74	82.2	1	33.3
Age	72.32	8.29	69	1.73
Race				
Hispanic/Latino	41	45.1	1	33.3
Black/African-American	29	31.9	1	33.3
Asian	8	8.8	0	0
White	6	6.6	1	33.3
Other	7	7.7	0	0
Marital status				
Married	21	23.3	1	33.3
Single	34	37.8	0	0
Divorced/separated	11	12.2	0	0
Widowed	24	26.7	2	66.7
Education				
Less than high school	18	20.2	0	0
Completed high school	25	28.1	1	33.3
Some college	26	29.2	1	33.3
Completed college	13	14.6	0	0
Graduate school	7	7.9	1	33.3
Income				
< $50,000	58	85.3	2	100.0
$50,000–$75,000	6	8.8	0	0
> $75,000	4	5.9	0	0

### UGAC in participants who provided urine samples

Of the 45 participants who provided urine samples, 19 samples had undetected urinary gallic acid concentrations (Supplemental Table 1). The values equating to zero were considered undetected. One of the participants with cancer prevalence had an undetected UGAC. Of the detected UGAC, two participants reported cancer prevalence. Overall, the mean gallic acid concentration among the participants with cancer (1.78 µg/mL) was much lower than the average UGAC among all the 45 participants (9.25 µg/mL).

### Gender differences in dietary behavior and UGAC

As shown in Table 4, female participants had significantly higher total daily intake of 100% juice (e.g., orange, apple, grape, or grapefruit juice), and higher total daily intake of fruit than male participants (*p* < 0.05). Additionally, female participants had a higher daily intake frequency of 100% juice and fruit, and a higher serving quantity of fruit than male participants (*p* < 0.05) (Tables 5 and 6). Furthermore, female participants had a higher daily intake frequency of other vegetables than male participants (*p* < 0.05). Moreover, female participants had a significantly higher UGAC than their male counterparts (*p* < 0.05) (Table 2).

**Table 2:** Associations among demographics, dietary behaviors, and gallic acid concentration (*N* = 91)

	Gallic acid concentration	*p* value
*Demographics*		
Age^1^	− 0.085	0.578
Gender^2^		**0.009**
Female	11.94	
Male	1.83	
Race^3^		0.775
White	1.31	
Black	11.78	
Hispanic/Latino	10.48	
Asian	0	
Other	2.49	
Material Status^2^		**0.08**
Married	1.06	
Other	12.26	
Education^2^		0.59
High school or below	7.80	
Some college or higher	10.76	
Income^2^		0.45
< $50,000	7.39	
≥ $50,000	2.36	
*Fruit and vegetable daily total intake*		
100% juice	0.10	0.518
Fruit	0.201	0.185
Lettuce salad	0.108	0.481
French fries or fried potatoes	− 0.114	0.461
Other white potatoes	− 0.115	0.452
Cooked dried beans	0.179	0.246
Other vegetables	0.139	0.375
Tomato sauce	− 0.101	0.514
Vegetable soup	0.012	0.937

The values in bold represent the values with statistical significance

^1^Pearson’s r, ^2^Independent ^3^ANOVA *t* test

### Dietary behavior differences in relation to age

While age was positively associated with fruit daily intake frequency and total daily intake (*p* < 0.05) and was marginally significantly associated with other vegetable daily intake frequency (*p* < 0.1) (Tables 4 and 5), age was later negatively associated with the serving quantity of French fries and other white potatoes (*p* < 0.05) (Table 6).

### Dietary behavior differences in relation to race/ethnicity

We found that race/ethnicity was marginally associated with total daily intake of French fries (*p* < 0.1) (Table 4). Specifically, Hispanic/Latino and Other participants had higher total daily intake of French fries compared to other race/ethnicity profiles. In addition, Asian participants had a higher daily intake frequency of fruit (*p* < 0.05) and higher daily intake frequency of vegetable soup compared to other racial/ethnic groups (*p* < 0.05) (Table 5).

### Dietary behavior and UGAC differences in relation to marital status

Marital status was significantly associated with the daily intake frequency of French fries (Tables 2, 3, 4, 5, and 6). While married participants had a lower daily intake frequency of French fries compared to other marital groups, (*p* < 0.05), they had higher daily intake frequency and total daily intake of vegetable soup (*p* < 0.05). In addition, although marital status was marginally associated with UGAC (*p* = 0.08), this association became significant (*p* < 0.05) after controlling for age, gender, education, and intake frequency of French fries.

**Table 3:** Associations among demographics, dietary behaviors, and gallic acid concentration (*N* = 91)

	Gallic acid concentration	*p* value
*Fruit and vegetable daily intake frequency*^1^		
100% juice	0.072	0.642
Fruit	0.086	0.576
Lettuce salad	0.081	0.596
French fries or fried potatoes	− 0.131	0.395
Other white potatoes	− 0.136	0.372
Cooked dried beans	0.085	0.579
Other vegetables	0.101	0.519
Tomato sauce	− 0.118	0.442
Vegetable soup	− 0.074	0.628
*Fruit and vegetable intake quantity*^2^		
100% juice	0.027	0.884
Fruit	0.246	0.112
Lettuce salad	0.046	0.777
French fries or fried potatoes	0.023	0.898
Other white potatoes	− 0.112	0.486
Cooked dried beans	0.166	0.305
Other vegetables	0.271	**0.083**
Tomato sauce	0.08	0.639
Vegetable soup	0.175	0.322

The values in bold represent the values with statistical significance

^1^Fruit and vegetable intake frequency refers to how often the participants’ intake fruit and vegetables was in the last month per day

^2^Fruit and vegetable intake quantity (cup equivalents) refers to how many/much fruit or vegetables the participants consumed each time

**Table 4:** Associations between demographics and fruit/vegetable daily total intake (*N* = 91)

	100% juice	Fruit	Lettuce salad	French fries/fried potatoes	Other white potatoes	Cooked dried beans	Other vegetables	Tomato sauce	Vegetable soup
Age^1^	0.01	0.21*	0.01	− 0.16	0.06 0	− 0.02	0.11	0.00	0.03
Gender^2^	*	***							
Female	**0.78**	**1.07**	0.53	0.03	0.13	0.20	0.74	0.10	0.14
Male	**0.18**	**0.37**	0.28	0.03	0.32	0.20	0.38	0.18	0.12
Race^3^				^**†**^					
White	0.15	0.77	0.21	**0.00**	0.14	0.19	0.77	0.08	0.04
Black	0.55	0.66	0.33	**0.02**	0.25	0.16	0.65	0.07	0.14
Hispanic/Latino	0.84	0.89	0.54	**0.04**	0.12	0.26	0.62	0.15	0.14
Asian	0.70	1.97	0.47	**0.01**	0.05	0.10	0.65	0.01	0.33
Other	0.67	1.68	0.90	**0.07**	0.14	0.13	0.96	0.22	0.10
Material status^2^									*
Married	0.32	0.97	0.57	0.01	0.13	0.21	0.44	0.11	**0.26**
Other	0.79	0.96	0.44	0.03	0.17	0.20	0.70	0.12	**0.12**
Education^2^						*			
High school or below	0.73	1.05	0.55	0.03	0.14	**0.28**	0.69	0.14	0.16
Some college or higher	0.62	0.88	0.41	0.03	0.16	**0.14**	0.63	0.07	0.14
Income^2^			*						^**†**^
< $50,000	0.66	0.82	**0.30**	0.03	0.20	0.19	0.67	0.13	**0.12**
≥ $50,000	0.49	1.17	**0.99**	0.04	0.08	0.31	0.50	0.05	**0.28**

The values in bold represent the values with statistical significance

^1^Pearson’s r, ^2^Independent *t* test, ^3^ANOVA

^†^*p* < 0.1, *p < .05, **p < .01, ***p < .001, two-tailed

^**^p < 0.01 This is a value for statistical significance. It is present in the notes of Table 4 to maintain the uniformity of the notes in Tables 4, 5, and 6

**Table 5:** Associations between demographics and fruit/vegetable daily intake frequency (*N* = 91)

	100% juice	Fruit	Lettuce salad	French fries/fried potatoes	Other white potatoes	Cooked dried beans	Other vegetables	Tomato sauce	Vegetable soup
Age^1^	0.08	0.25*	0.08	− 0.06	0.11 0	0.00	**0.21**^**†**^	0.11	0.15
Gender^2^	**	*					*		
Female	**0.67**	**1.24**	0.69	0.11	0.19	0.27	**0.73**	0.17	0.17
Male	**0.21**	**0.68**	0.38	0.13	0.31	0.30	**0.41**	0.32	0.16
Race^3^		**							**
White	0.21	**0.89**	0.44	0.02	0.17	0.31	1.02	0.18	**0.07**
Black	0.60	**0.89**	0.55	0.07	0.31	0.24	0.68	0.13	**0.15**
Hispanic/Latino	0.60	**0.96**	0.73	0.16	0.18	0.34	0.57	0.27	**0.15**
Asian	0.72	**2.60**	0.42	0.03	0.08	0.36	0.93	0.03	**0.47**
Other	0.54	**1.93**	0.77	0.13	0.17	0.17	0.79	0.22	**0.09**
Material status^2^				*					*
Married	0.36	1.26	0.62	**0.05**	0.19	0.38	0.50	0.16	**0.28**
Other	0.66	1.09	0.63	**0.12**	0.22	0.27	0.71	0.21	**0.14**
Education^2^						*		*	
High school or below	0.49	1.18	0.71	0.13	0.21	**0.40**	0.64	**0.28**	0.18
Some college or higher	0.63	1.13	0.56	0.09	0.20	**0.21**	0.70	**0.10**	0.17
Income^2^			*						
< $50,000	0.50	1.06	**0.50**	0.12	0.25	0.31	0.68	0.23	0.15
≥ $50,000	0.51	1.26	**1.13**	0.15	0.12	0.41	0.56	0.15	0.26

The values in bold represent the values with statistical significance

^1^Pearson’s r, ^2^Independent *t* test, ^3^ANOVA

^†^*p* < 0.1, *p < .05, **p < .01, ***p < .001, two-tailed

^**^p < 0.001 This is a value of statistical significance. It is included in the notes of Tables 5 and 6 to maintain the uniformity of the notes in Tables 4, 5, and 6

**Table 6:** Associations between demographics and fruit/vegetable serving quantity (*N* = 91)

	100% juice	Fruit	Lettuce salad	French fries/fried potatoes	Other white potatoes	Cooked dried beans	Other vegetables	Tomato sauce	Vegetable soup
Age^1^	− 0.21	0.02	− 0.17	− 0.35**	− 0.23* 0	− 0.18	− 0.06	− 0.13	− 0.16
Gender^2^		*							
Female	0.92	**0.76**	0.48	0.28	0.69	0.67	0.86	0.45	0.79
Male	0.98	**0.57**	0.64	0.26	0.80	0.69	0.77	0.52	0.87
Race^3^									
White	0.91	0.75	0.42	0.20	0.82	0.75	0.75	0.40	0.72
Black	0.84	0.71	0.60	0.26	0.66	0.60	0.86	0.43	0.85
Hispanic/Latino	1.03	0.73	0.62	0.29	0.74	0.72	0.82	0.46	0.80
Asian	0.75	0.75	0.78	0.20	0.74	0.42	0.72	0.33	0.78
Other	1.04	0.82	0.75	0.37	0.74	0.75	1.13	0.80	1.13
Material status^2^									
Married	0.81	0.74	0.70	0.28	0.68	0.62	0.85	0.49	0.87
Other	0.96	0.75	0.60	0.27	0.73	0.68	0.82	0.46	0.81
Education^2^									
High school or below	1.06	0.70	0.58	0.25	0.72	0.65	0.81	0.46	0.80
Some college or higher	0.85	0.76	0.67	0.31	0.71	0.68	0.85	0.45	0.85
Income^2^									
< $50,000	0.99	0.74	0.55	0.25	0.73	0.62	0.81	0.44	0.78
≥ $50,000	0.83	0.73	0.85	0.34	0.72	0.75	0.81	0.35	0.90

^1^Pearson’s r, ^2^Independent *t* test, ^3^ANOVA

^†^*p* < 0.1, *p < .05, **p < .01, ***p < .001, two-tailed

^†^This value shows marginal significance. It is included in the notes of Table 6 to maintain uniformity among the notes in tables 4, 5, and 6

^**^p < 0.001 This is a value of statistical significance. It is included in the notes of Tables 5 and 6 to maintain the uniformity of the notes in Tables 4, 5, and 6

### Dietary differences in relation to educational attainment

Participants with high school or lower education had significantly higher daily intake frequency and total daily intake of cooked dried beans (*p* < 0.05), and higher daily intake frequency of tomato sauce compared to other education levels (*p* < 0.05) (Tables 4 and 5).

### Dietary differences in relation to income

We found that higher income was significantly associated with higher total daily intake and daily intake frequency of lettuce salad (*p* < 0.05), and it was also marginally significantly associated with total daily intake of vegetable soup (*p* < 0.1) (Tables 4 and 5).

### Multivariate analysis between dietary behavior and UGAC

Associations between UGAC and certain dietary behaviors became significant after controlling for demographic variables (Supplemental Table 2). The association between daily intake frequency of dried beans and UGAC became marginally significant (*p* < 0.1) after controlling for income, which indicated that people who have higher daily intake frequency of dried beans are more likely to have higher UGAC. The serving quantity of other vegetables became significantly (*p* < 0.05) associated with UGAC after controlling for marital status, and the association between serving quantity of fruit and UGAC also became significant (*p* < 0.05) after controlling for age, gender, marital status, education, and income, indicating that participants with a higher serving quantity of other vegetables and fruits are more likely than others to have higher UGAC level.

## Discussion

This study has found that there are significant dietary behavior differences in the diverse neighborhood of East Harlem, New York City.

We observed significant dietary associations based on age. Older participants had a significantly higher total daily intake of fruits and frequency intake of vegetables, while they had a significantly lower serving quantity of French fries and other white potatoes. This may explain the rising incidence of colorectal cancer at a younger age [33]. We also saw that Hispanic/Latino participants, along with those identifying as Other, had a higher intake of French fries, while Asian participants had significantly higher fruit intake and vegetable soup intake frequencies than other racial groups. This may explain the increasing incidence of early onset colorectal cancer in Hispanics [34]. While it has been shown that Blacks/African-Americans have a higher incidence and mortality rate of cancers compared to other racial/ethnic groups in the United States, we did not observe significant dietary behavior associations with Black/African-American participants. Future studies will need to be performed to further explore the potential role of dietary behavior in cancer risk among Blacks/African-Americans.

We also observed particular dietary behaviors related to income. Although most participants reported an annual income lower than $50,000, participants with a higher income had a higher total daily intake and daily higher frequency of intake of salad. Although from the same district, people with higher income had a dietary behavior reflective of a healthier diet. Previous work has shown that healthier food options are limited by access due to food prices [35]. As such, these are factors that can contribute to cancer disparities.

After controlling for marital status, other vegetable serving quantity was significantly associated with UGAC. That is, among participants reporting the same marital status, those with a higher quantity of vegetable intake were more likely to have higher UGAC levels. Furthermore, after controlling for age, gender, marital status, education, and income, the association between fruit serving quantity and UGAC was also significant. That is, among participants with the same demographic characteristics, those with a higher fruit intake quantity are more likely to have a higher UGAC. These results validate the presence of gallic acid in various fruits and vegetables, as previous studies have shown [36]. Additionally, they demonstrate the utility of UGAC as a biomarker of a healthy diet.

In the present study, only two male participants and one female participant reported having cancer. All of them had income lower than $50,000, and two of them were widowed. Additionally, only two out of the three participants with cancer had detectable UGAC. Notably, the UGAC in these participants with cancer was lower in comparison to the UGAC from most of all the studied samples. Although these findings are not generalizable, they may be reflective of known factors of cancer risk. Furthermore, the fact that all participants with cancer had either no detectable UGAC or low detectable UGAC appears to be in agreement with the higher risk of cancer among people with a diet low in fruits and vegetables.

Our study has several limitations. The sample size of 91 participants was relatively small. Furthermore, only 45 participants provided urine samples. Still, we found meaningful associations between dietary intake and UGAC within our study population. Additionally, we did not collect information regarding alcohol consumption, smoking, and current or previous occupations of participants. Another limitation is the use of a FFQ for assessment of dietary behavior. As a self-reported survey, the FFQ may include some bias and may not account for some cultural differences. Furthermore, we used gallic acid as the only nutritional biomarker due to its anticancer properties and its prevalence in fruits and vegetables. However, we acknowledge the spectrum of existing nutritional biomarkers and the possibility of using other metabolites as nutritional biomarkers. We will consider these limitations in future studies and incorporate a more comprehensive questionnaire for data collection. Nevertheless, our findings have meaningful implications for recognizing dietary behavior differences that can impact cancer prevention strategies with implications for cancer health disparities.

This study provides meaningful information on dietary behavior in a diverse community in New York City. While we observed some statistically significant differences in dietary behavior based on demographics such as gender, race/ethnicity, income, and age, additional larger studies are warranted to confirm the generalizability of our findings to minority groups in other cities, and to investigate the non-significant associations uncovered in our study. Future research should investigate additional confounding risk factors that have significant implications for cancer prevention.

## Supplementary Information

Below is the link to the electronic supplementary material.

Supplementary file1 (DOCX 16 KB)

## Data Availability

Enquiries about data availability should be directed to the authors.

## References

[1] Siegel RL (2020). Cancer statistics, 2020. CA Cancer J Clin.

[2] Gardner K (2018). The science of cancer health disparities: a young discipline with an old heritage. Am J Pathol.

[3] American Cancer Society (2019). Cancer facts & figures for African Americans 2019–2021.

[4] Stefanidis D (2006). Colorectal cancer in Hispanics: a population at risk for earlier onset, advanced disease, and decreased survival. Am J Clin Oncol.

[5] Kim HI (2018). Sex differences in cancer: epidemiology, genetics and therapy. Biomol Ther (Seoul).

[6] O'Connor JM (2018). Factors associated with cancer disparities among low-, medium, and high-income US counties. JAMA Netw Open.

[7] White A (2018). A review of sex-related differences in colorectal cancer incidence, screening uptake, routes to diagnosis, cancer stage and survival in the UK. BMC Cancer.

[8] Lou L (2020). Sex difference in incidence of gastric cancer: an international comparative study based on the Global Burden of Disease Study 2017. BMJ Open.

[9] Kohi MP (2016). Gender-related differences in hepatocellular carcinoma: does sex matter?. J Vasc Interv Radiol.

[10] Ansari D (2019). Early-onset pancreatic cancer: a population-based study using the SEER registry. Langenbecks Arch Surg.

[11] Sheh A (2011). 17beta-estradiol and tamoxifen prevent gastric cancer by modulating leukocyte recruitment and oncogenic pathways in Helicobacter pylori-infected INS-GAS male mice. Cancer Prev Res (Phila).

[12] Shin JY (2019). Molecular markers in sex differences in cancer. Toxicol Res.

[13] Manne U (2016). Molecular biomarkers of colorectal cancer and cancer disparities: current status and perspective. Curr Colorectal Cancer Rep.

[14] Gonzalez-Pons M (2019). Abstract 740: mutational landscape of early-onset colorectal cancer tumors in Hispanics. Can Res.

[15] Key TJ (2004). Diet, nutrition and the prevention of cancer. Public Health Nutr.

[16] Thanikachalam K, Khan G (2019). Colorectal cancer and nutrition. Nutrients.

[17] Schütte K (2016). Nutrition and hepatocellular cancer. Gastrointest Tumors.

[18] Stojcev Z (2013). The role of dietary nutrition in stomach cancer. Contemp Oncol (Pozn).

[19] Benham V, Bernard JJ (2018). Why does a high-fat diet increase cancer risk?. Future Oncol.

[20] Kunzmann AT (2015). Dietary fiber intake and risk of colorectal cancer and incident and recurrent adenoma in the prostate, lung, colorectal, and ovarian cancer screening trial. Am J Clin Nutr.

[21] Sauvageot N (2013). Use of food frequency questionnaire to assess relationships between dietary habits and cardiovascular risk factors in NESCAV study: validation with biomarkers. Nutr J.

[22] Mennen LI (2006). Urinary flavonoids and phenolic acids as biomarkers of intake for polyphenol-rich foods. Br J Nutr.

[23] Verma S (2013). Gallic acid: molecular rival of cancer. Environ Toxicol Pharmacol.

[24] Badhani B (2015). Gallic acid: a versatile antioxidant with promising therapeutic and industrial applications. RSC Adv.

[25] Gao J (2019). A role of gallic acid in oxidative damage diseases: a comprehensive review. Nat Product Commun.

[26] Volf I (2014). Thermal stability, antioxidant activity, and photo-oxidation of natural polyphenols. Chem Pap.

[27] Holman DM, White MC (2011). Dietary behaviors related to cancer prevention among pre-adolescents and adolescents: the gap between recommendations and reality. Nutr J.

[28] Uauy R, Solomons N (2005). Diet, nutrition, and the life-course approach to cancer prevention. J Nutr.

[29] Presley CJ (2016). Gaps in nutritional research among older adults with cancer. J Geriatr Oncol.

[30] Zavala VA (2020). Cancer health disparities in racial/ethnic minorities in the United States. Br J Cancer.

[31] Thompson FE (2002). Fruit and vegetable assessment: performance of 2 new short instruments and a food frequency questionnaire. J Am Diet Assoc.

[32] Zambrano CN (2020). Dietary behavior and urinary gallic acid concentrations in older minority residents of East Harlem, New York City. J Racial Ethn Health Dispar.

[33] Bhandari A (2017). Colorectal cancer is a leading cause of cancer incidence and mortality among adults younger than 50 years in the USA: a SEER-based analysis with comparison to other young-onset cancers. J Investig Med.

[34] Koblinski J (2018). Disparities in incidence of early- and late-onset colorectal cancer between Hispanics and Whites: a 10-year SEER database study. Am J Surg.

[35] Drewnowski A, Eichelsdoerfer P (2010). Can low-income Americans afford a healthy diet?. Nutr Today.

[36] Singh JP (2016). Composition, bioactive compounds and antioxidant activity of common Indian fruits and vegetables. J Food Sci Technol.

