# Supplementary material for: Dietary behavior and urinary gallic acid concentration differences among underserved elder racial and ethnic minorities in New York City
Source: Cancer Causes Control. Author manuscript; Available in PMC 2022 Jul 8. (PMC9188520; doi:10.1007/s10552-022-01581-y)
Supplement: Supp [file NIHMS1800068-supplement-Supp.docx]

**Supplemental Table 1.** Urinary gallic acid concentration among all participants and among cancer cases

| Urinary gallic acid concentration (µg/mL) in different groups | N | Range | Minimum | Maximum | Mean | Standard Deviation |
| --- | --- | --- | --- | --- | --- | --- |
| In ALL cases | 45 | 88.97 | 0.00 (undetected) | 88.97 | 9.25 | 18.14 |
| In cancer cases | 2 | 0.11 | 1.73 | 1.84 | 1.78 | 0.08 |

Note: 42.2% (n=19) of participants had undetectable urinary gallic acid

**Supplemental Table 2.** Regression on Gallic Acid Concentration by Demographics and Dietary Behaviors (N=45)

|  | | Model 1 | Model 2 | Model 3 | Model 4 |
| --- | --- | --- | --- | --- | --- |
| Constant | | 14.48 | 4.15 | -6.20 | -16.74 |
| Age | | -0.19 |  |  | 0.09 |
| Female | |  |  |  | 3.21 |
| Not in married status | | 13.17* |  | 9.73* | 6.94 |
| Education | | 0.48 |  |  |  |
| Income | |  | -3.42 |  | -3.26 |
| French Fries/fried potatoes daily intake frequency | | -14.84 |  |  |  |
| Dried bean daily intake frequency | |  | 9.17^†^ |  |  |
| Other vegetable serving quantity | |  |  | 8.19* |  |
| Fruit serving quantity | |  |  |  | 15.75* |
|  | |  |  |  |  |
| R^2^ | 0.117 | | 0.087 | 0.184 | 0.253 |

*Note:* *^†^p<0.1; *p < .05; **p < .01; ***p < .001, two-tailed*
